# Occupational respiratory symptoms and associated factors among street sweepers in low- and middle-income countries: A systematic review and meta-analysis

**DOI:** 10.1371/journal.pone.0320237

**Published:** 2025-04-09

**Authors:** Belay Desye, Abebe Kassa Geto, Leykun Berhanu, Chala Daba, Gete Berihun

**Affiliations:** 1 Department of Environmental Health, College of Medicine and Health Sciences, Wollo University, Dessie, Ethiopia; 2 Department of Public Health, College of Medicine and Health Sciences, Woldia University, Woldia, Ethiopia; 3 National Center for Epidemiology and Population Health, The Australia National University, Canberra, ACT, Australia; 4 Department of Environmental Health, College of Medicine and Health Sciences, Debre Markos University, Debre Markos, Ethiopia; Visva-Bharati University: Visva-Bharati, INDIA

## Abstract

In low- and middle-income countries, occupational exposure continues to be a serious public health concern. Because of their working conditions, street sweepers are particularly vulnerable to health hazards, with respiratory issues being the most common. The lack of comprehensive and inconsistence evidence on occupational respiratory symptoms among street sweepers, which exacerbates this issue. This study aimed to estimate the pooled prevalence of occupational respiratory symptoms and associated factors among street sweepers in low- and middle-income countries. A comprehensive search strategy was carried out using various databases, such as PubMed, Scientific Direct, HINARI and Google Scholar, and the grey literature. The preferred reporting items for systematic reviews and meta-analyses were used. Microsoft Excel was used to extract the data, which was then transferred to STATA 14/SE software for analysis. The Joanna Briggs Institutes’ quality appraisal tool was used to ensure the quality of the included articles. A random effects model was used to estimate the pooled prevalence. In this study, a subgroup analysis was employed to examine the potential sources of heterogeneity, and sensitivity analysis was conducted to assess the effect of individual studies on the pooled results. To evaluate publication bias, the funnel plot and Egger’s regression tests were employed. The pooled prevalence of occupational respiratory symptoms among street sweepers was estimated to be 47.35% (95% CI: 34.59–60.11). Among each respiratory symptom, cough was found to be the most prevalent at 38.37% (95% CI: 28.54–48.19). In this study, past dust exposure (OR = 2.84, 95% CI: 1.06–4.61), not using facemasks (OR = 2.67, 95% CI: 1.54–3.81), having more than five years of work experience (OR = 3.68, 95% CI: 1.66–5.7), and being between the ages of 38 and 47 (OR = 2.17, 95% CI: 1.07–3.27) were found to be predictor factors with occupational respiratory symptoms among street sweepers. Furthermore, there is no evidence of publication bias for this study. The findings of this study indicated a significant prevalence of occupational respiratory symptoms among street sweepers. Therefore, it is highly recommended to focus on the provision and proper use of personal protective equipment and to enhance occupational health and safety training.

## 1. Introduction

Globally, occupational respiratory diseases are a serious public health concern to workers [1,2]. An estimate of 2.78 million workers died from occupational accidents and disease annually worldwide, reported in 2017. Occupational respiratory disorders account for up to 30% of all documented work-related deaths, and employees in high-risk industries including mining, construction, and dust-generating activities are 50% more likely to suffer from occupational respiratory illnesses [3].

Respiratory symptoms such as cough, phlegm, wheezing, difficulty breathing, and chest pain are indicative of respiratory problems that are mainly developed as the result of workplace exposures [4]. Inhaling dust can trigger inflammatory responses in the respiratory system [5,6]. Studies on exposure to organic dust suggest that occupational respiratory disease development is influenced in part by the interaction of dust particles with antibodies in the respiratory lining, leading to an immune response that may result in conditions such as asthma (the most prevalent respiratory disease), chronic obstructive pulmonary diseases, silicosis, or pulmonary arterial hypertension [7,8].

Street sweepers are essential to protecting the environment and public health. However, they are exposed to a number of occupational health problems due to the nature of their occupation, primarily respiratory issues [9]. Street sweepers often reside in inadequate housing with little attention paid to their health, have lower educational attainment, and come from low socioeconomic backgrounds [10,11]. Soils, sand, gravel, dust particles, vehicle dust, bio-aerosols, and plant particles are among the harmful dusts they are exposed to in the atmosphere [12].

Standardized waste management procedures, such as the frequent use of covered containers for garbage collection, greatly reduce occupational health issues among street sweepers in many developed nations [13]. On the other hand, waste is handled improperly in Low and Middle Income Countries (LMICs), where it is frequently disposed of in open landfills rather than in closed storage containers [14].

The prevalence of respiratory problems among street sweepers ranged from 14.83% in Nigeria [15] and 70.3% in Ethiopia [16]. Evidence suggests a number of characteristics that have been linked to respiratory symptoms, including age, work experience, educational attainment, history of diseases, failure to wear a facemask, and prior dust exposure [16–21]. However, inconsistencies were found in the prevalence levels of street sweeper respiratory symptoms, and the statistical significance of associated factors varied accordingly. This underscores the need for effective interventions and compressive evidence.

Street sweepers in LMICs are exposed to serious health risks such as dust, noise, and biological agents, which can cause respiratory problems and musculoskeletal diseases. It is crucial to investigate occupational symptoms among these workers [18,22]. Due to financial and awareness conditions, many street sweepers do not have enough Personal Protective Equipment (PPE), which increases their risk of health issues [18,19,22]. The well-being of this workforce is critical not only for their own health but also for public health, as poor health outcomes can affect urban sanitation and increase healthcare costs. This study can inform stakeholders and policymakers, promoting better health care and working conditions, which will eventually benefit employees and the communities they serve.

Furthermore, this study is crucial for prioritize the health and safety of workers and contributing to the United Nations 2030 Agenda for Sustainable Development Goals (SDGs), particularly SDG 3, which aims to “ensure healthy lives and promote well-being,” and SDG 8, which focuses on “decent work” for all. [23]. Therefore, the aim of this study is to estimate the pooled prevalence of occupational respiratory symptoms and to identify predictor factors among street sweepers in LMICs.

## 2. Methodology

### 2.1. Study setting and registration of protocols

**Fig 1:**
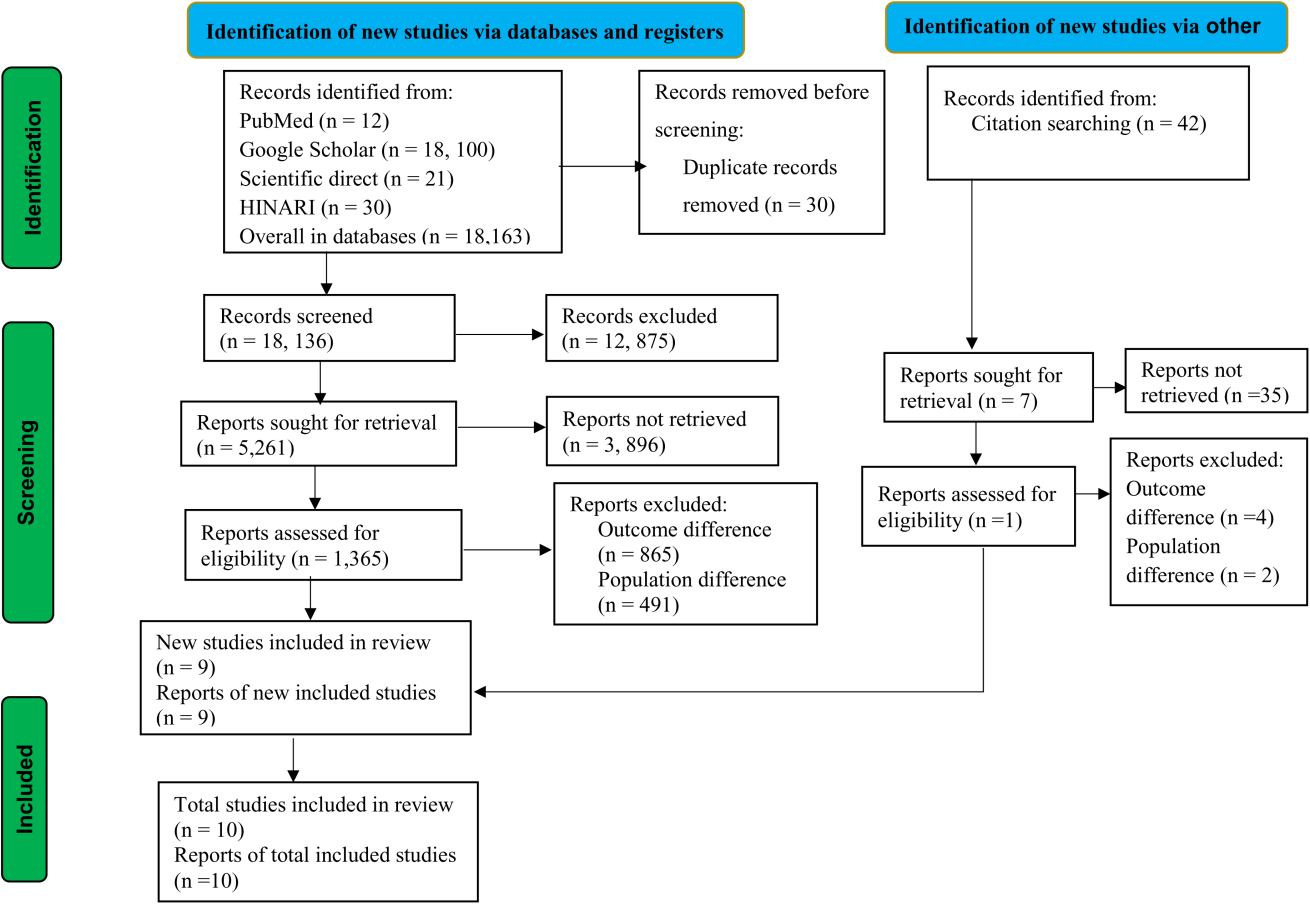
PRISMA flow diagram of this study, 2024.

The guidelines for updated Preferred Reporting Items for Systematic Reviews and Meta-Analysis (PRISMA) were used for this study [24] (Fig 1). The review protocol for this study was registered in the International Prospective Register of Systematic Reviews (PROSPERO) with the record ID CRD42024545517. This study was conducted in LMICs, following the list of World Bank data [25].

### 2.2. Sources of information and search strategies

A systematic literature search was undertaken using a database of PubMed, Scientific Direct, Google Scholar, and HINARI. The search was conducted until June 10, 2024. For example, for the PubMed search, key terms were used in combination with the Boolean operators “AND” and “OR”. Apart from conducting an electronic database search, additional articles were obtained by searching the gray literature through direct Google searches and by examining the bibliographies of the included articles. In this study we have included the details of the searching strategies (S1 File).

### 2.3. Eligibility criteria

Inclusion criteria: This review included studies that fulfilled the following criteria.

- **Population**: street sweepers
- **Outcomes**: studies assessed the respiratory symptoms of street sweepers and identified factors.
- **Study Design**: all observational studies (cross-sectional, cohort, and case control).
- **Study setting**: studies conducted in LMICs.
- **Language of published articles**: articles written in English
- **Publication condition**: Both published and unpublished articles were included.

Exclusion criteria: studies that were not fully accessible despite three personal email contacts with the primary and corresponding authors, and studies that did not clearly show us the outcome interest of the study were excluded. Furthermore, certain types of research articles, including letters to editors, qualitative studies, systematic reviews, short communications, and commentaries, were not considered.

### 2.4. Operational definition

Occupational respiratory symptoms: the development of one or more of the respiratory symptoms, such as cough, phlegm, wheezing, breathlessness, and chest tightness, among workers [26,27].

### 2.5. Study selection

Two investigators, BD and GB, conducted independent screenings of articles based on their titles, abstracts, and full texts to identify eligible articles. They followed pre-established inclusion and exclusion criteria during this process. The screened articles were then combined by the two investigators, and any disagreements that arise during data abstraction and selection were resolved through evidence-based discussions and the involvement of a third investigator (CD). A total of 1362 articles were excluded due to outcome and population difference (S2 File).

### 2.6. Data extraction and management

The data extraction format used in this study included the name of the author, publication year, study country, sample size, symptoms, prevalence, and risk of bias. These details were organized in a table format (Table 1). To collect articles and remove duplicate studies, Zotero Reference Manager was used. Additionally, the updated PRISMA checklist was employed to effectively summarize the study conditions [24] (S3 File).

**Table 1:** Major characteristics of the included articles in occupational respiratory symptoms among street sweepers in LMICs, 2024.

Reference	Study country	Sample size	Symptoms	Prevalence (%)	Risk of Bias
Worede et al. [17]	Ethiopia	391	138	35.29	Low
Tamene et al. [18]	Ethiopia	405	279	68.89	Low
Manaye et al. [19]	Ethiopia	392	178	45.41	Low
Wubet [16]	Ethiopia	570	400.71	70.3	Low
Beyene [21]	Ethiopia	208	113	54.33	Low
Eneyew et al. [20]	Ethiopia	84	41	48.81	Low
Stambuli [30]	Tanzania	102	43.83	42.971	Low
Mostafa et al. [31]	Egypt	107	41.9	39.16	Moderate
Habybabady et al. [32]	Iran	84	44.8	53.33	Moderate
Nku et al. [15]	Nigeria	200	29.66	14.83	Moderate

### 2.7. Quality assessment of the studies

The quality appraisal tools of the Joanna Briggs Institute (JBI) for an analytical cross-sectional study were used to assess the quality of the included studies [28]. In addition to avoid missing of articles, we had rigorously searched using different databases and search strategies. The quality of the articles was independently assessed by the two reviewers (BD and GB). Eight criteria were used to assess the quality of each article. The assessment options were categorized as yes, no, unclear, or not applicable. The risk of bias was classified as low (total score between 6–8), moderate (total score between 3–5), and high (total score between 0–2). Lastly, only studies categorize under low and moderate risks of biases were included in this review (S4 File).

### 2.8. Outcome of interest

This study has three main outcomes:

- The pooled prevalence of occupational respiratory symptoms among street sweepers, expressed as (%)
- The pooled prevalence of each respiratory symptom among street sweepers, expressed as (%)
- The pooled AOR of predictor factors associated with occupational respiratory symptoms among street sweepers in LMICs, expressed as (AOR)

### 2.9. Statistical methods and data analysis

Microsoft Excel was used to extract data, which was then transported to STATA version 14 for analysis. The index of heterogeneity (*I²* statistic) was employed to evaluate the heterogeneity among the included articles, with values of 25%–50%, 50%–75%, and greater than 75% indicating low, moderate, and high heterogeneity, respectively [29]. The random effect model was used to estimate the pooled prevalence of the study.

To investigate potential variations, a subgroup analysis was performed based on study countries, sample sizes, and publication years. Sensitivity analysis was conducted to evaluate the effect of each study on the estimated pooled results. A funnel plot test and Egger’s regression test with a significance level of *p < 0.05* as the cut point were used to ensure the presence of publication bias. To identify possible sources of heterogeneity, univariate meta-regression was employed. Finally, the findings of this study were presented through tables, figures, a forest plot, and descriptive texts.

## 3. Results

### 3.1. Overview of the search process

In this review, using a database and grey literatures of search, a total of 18,205 articles were identified. After duplicate records were removed, 18,136 records were screened for this review. Following the records, only 5,261 reports were sought for retrieval. After being identified for retrieval, 1,365 reports were evaluated for eligibility. Following eligibility, a total of 1,356 studies were excluded due to differences in outcome interest and population differences. Finally, a total of 9 studies were included in this study from database sources. In addition to the database sources, 1 study was included in this review from other sources (grey literature and citation searching). As presented in the PRISMA flowchart, a total of 10 studies were included in the meta-analysis (Fig 1).

### 3.2. Characteristics of the eligible studies

All the included studies were cross-sectional studies. For the estimation of the pooled prevalence of respiratory symptoms among street sweepers, a total of 2,543 sample sizes were included. Most of the studies were conducted in Ethiopia (n = 6), followed by Tanzania (n = 1), Egypt (n = 1), Iran (n = 1) and Nigeria (n = 1). The included studies were conducted between 2005 and 2022. In this study, a total of 1,310 respiratory symptoms among street sweepers were observed. The prevalence of respiratory symptoms in this review ranged from 14.83% in Nigeria [15] to 70.3% in Ethiopia [16]. All the included articles were categorized under low and moderate levels of risk of bias (Table 1).

### 3.3. The pooled prevalence of occupational respiratory symptoms among street sweepers in LMICs

**Fig 2:**
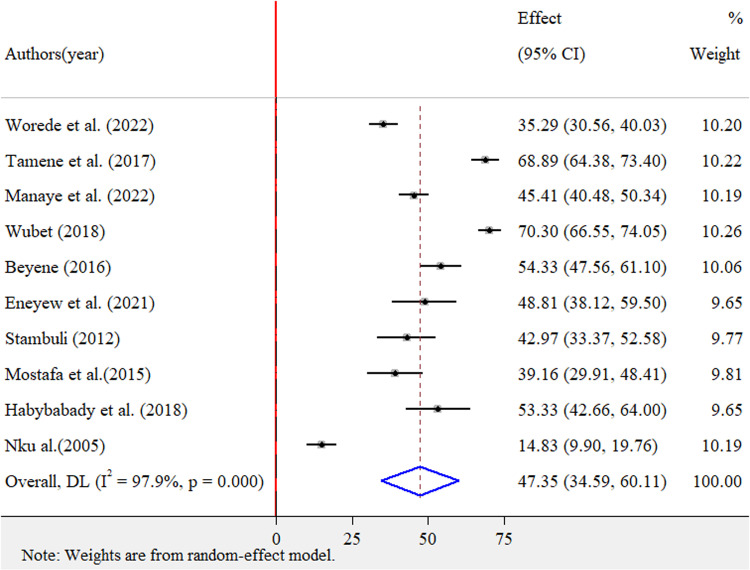
Forest plot for pooled prevalence of occupational respiratory symptoms among street sweepers in LMICs, 2024.

The pooled prevalence of occupational respiratory symptoms using a random-effect model was estimated at 47.35% (95% CI: 34.59–60.11), with high heterogeneity (*I*^2^ = 97.9%, p-value <0.001) (Fig 2). In addition, among each respiratory symptom, cough at 38.37% (95% CI: 28.54–48.19) and breathlessness at 29.82% (95% CI: 19.17–40.46) were found to be relatively higher (Table 2).

**Table 2:** Pooled prevalence of respiratory symptoms among street sweepers in LMICs, 2024.

Respiratory symptoms	Number of studies	Pooled prevalence	*I*^*2* (^%)	*p-value*
Cough	10	38.37% (95% CI: 28.54–48.19)	96.9	<0.001
Phlegm	8	24.56% (95% CI: 20.11–29.02)	83.2	<0.001
Wheezing	9	24.76% (95% CI: 15–34.51)	98	<0.001
Chest tightness	3	18.24% (95% CI: 15.10–21.38)	0.0	0.988
Breathlessness	5	29.82% (95% CI: 19.17–40.46)	95.7	<0.001

A sub-group analysis based on study countries, year of publication, and sample size was performed to examine the potential sources of heterogeneity. The highest pooled prevalence of 53.94% (95% CI: 41.21–66.68) was observed in Ethiopia, and the lowest pooled prevalence was estimated at 36.21% (95% CI: 31.6–40.82) in Nigeria. Heterogeneity was highest among studies conducted in Ethiopia *(I*^*2*^ *= 97.2%).*

A subgroup analysis based on the year of publication, a study conducted from 2015–2020 was 57.88% (*95% CI:* 47.85–67.91) with *(I*^*2*^ *= 92.7%, p-value <0.001)* found to be higher, and a study conducted from <2010 was 14.8% (95% CI: 9.90–19.76) with *(I*^*2*^ *= 0.0%, p-value <0.001)* found to be lower. In addition, a subgroup analysis based on sample size, a study conducted with >400 was 69.72% (95% CI: 66.84–72.61) with *(I*^*2*^ *= 0.0%, p-value = 0.637)* found to be higher, and a study conducted with a sample size of 200–300 was 34.51*%* (95% CI: -*4.2*–73.21) with *(I*^*2*^ *= 98.8%, p-value <0.001)* found to be lower (Table 3).

**Table 3:** Subgroup analysis of the pooled prevalence of occupational respiratory symptoms among street sweepers in LMICs, 2024.

Subgroup criteria	Number of studies	Pooled prevalence	Heterogeneity	
*I* ^*2*^ *(%)*	*p-value*
Study country				
Ethiopia	6	53.94% (95% CI: 41.21–66.68)	97.2	<0.001
Tanzania	1	42.97% (95% CI: 33.37–52.58)	0.0	<0.001
Egypt	1	39.16% (95% CI: 29.91–48.41)	0.0	<0.001
Iran	1	40.31% (95% CI: 12.60–68.01)	0.0	<0.001
Nigeria	1	36.21% (95% CI: 31.6–40.82)	0.0	<0.001
Year of Publication				
>2020	3	42.45% (95% CI: 34.18–50.71)	81.3	0.005
2015-2020	5	57.88% (95% CI: 47.85–67.91)	92.7	<0.001
2010-2015	1	42.97% (95% CI: 33.37–52.58)	0.0	<0.001
<2010	1	14.83% (95% CI: 9.90–19.76)	0.0	<0.001
Sample size				
>400	2	69.33% (95% CI: 66.84–72.61)	0.0	0.637
300-400	2	40.33% (95% CI: 30.42–50.24)	88.1	0.004
200-300	2	34.51% (95% CI: -4.20–73.21)	98.8	<0.001
<200	4	45.63%(95% CI: 39.49–51.77)	33.5	0.211

In this study, to identify the potential source of heterogeneity, a univariate meta-regression was conducted using study country, sample size, and study year as factors to identify the source of heterogeneity. However, neither of them was found to be statistically significant as sources of heterogeneity (Table 4).

**Table 4:** Univariate meta-regression of occupational respiratory symptoms among street sweepers in LMICs, 2024.

Variable	Coefficient	p-value	95% CI
Publication year	0.0017	0.957	(−0.72–0.75)
Sample size	0.01	0.932	(−0.29–0.31)
Study country	−0.19	0.897	(−3.67–3.28)
Constant	3.55	0.699	(−17.84–24.95)

**Fig 3:**
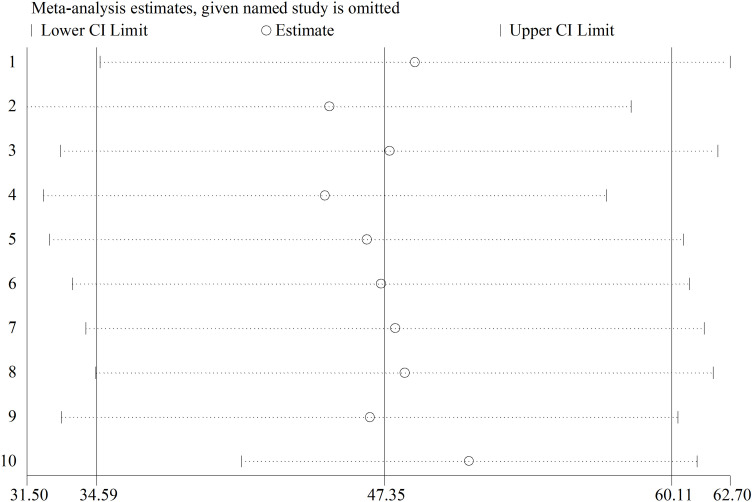
A sensitivity analysis of occupational respiratory symptoms among street sweepers in LMICs, 2024.

**Fig 4:**
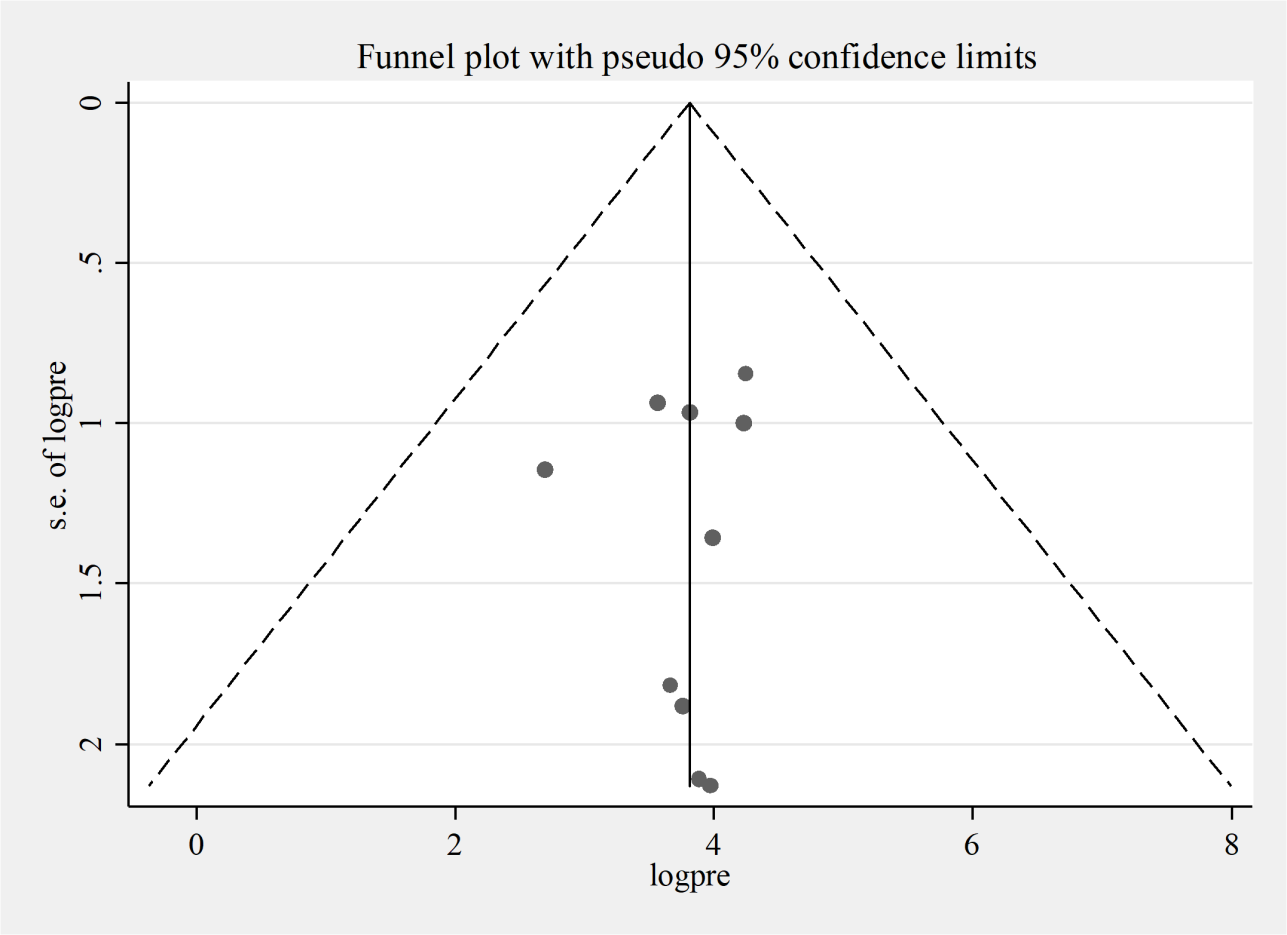
Funnel plot of occupational respiratory symptoms among street sweepers in LMICs, 2024.

A sensitivity analysis was conducted to evaluate the effect of a single study. The findings revealed that there is no strong evidence for the effect of a single study (Fig 3). In addition, the funnel plot showed as symmetrically distributed as visualized, implying no evidence for the publication bias (Fig 4). In addition to the funnel plot, the Egger-regression test confirmed that there is no publication bias for this study (*p-value = 0.723*).

### 3.4. Factors associated with the occupational respiratory symptoms among street sweepers

**Fig 5:**
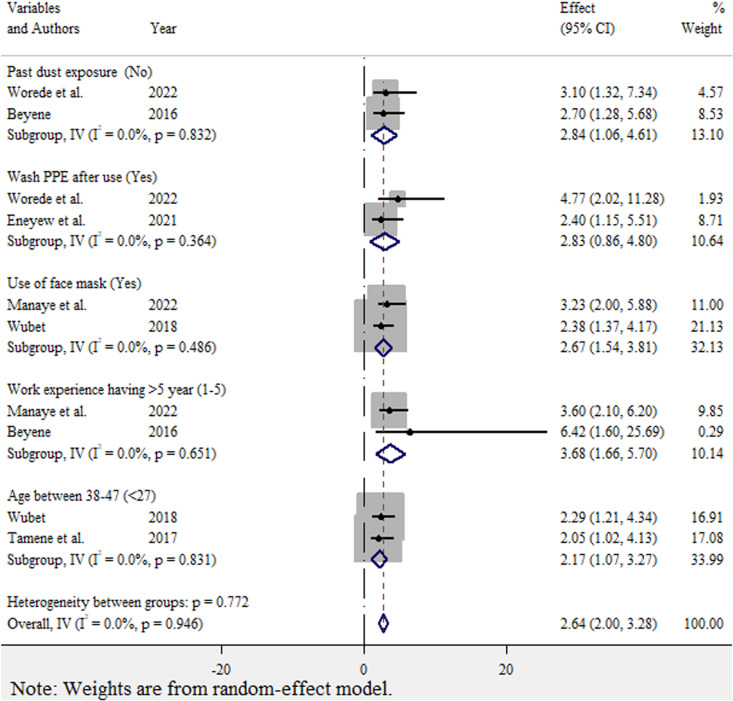
Forest plot for factors affecting occupational respiratory symptoms among street sweepers in LMICs, 2024.

Factors associated with occupational respiratory symptoms among street sweepers were assessed using 6 studies [16–21]. Among the 6 studies, the findings of 2 studies [17,21] reported that street sweepers with past dust exposure were 2.84 times more likely to develop respiratory symptoms than those without past dust exposure (OR = 2.84, 95% CI: 1.06–4.61). In this study, 2 studies [16,19] revealed that street sweepers who did not use facemasks were 2.67 times more likely to develop respiratory symptoms than who did use facemasks (OR = 2.67, 95% CI: 1.54–3.81). According to 2 studies [19,21], street sweepers who have more than five years of work experience were more likely to develop respiratory symptoms than street sweepers who have experience from 1–5 years (OR = 3.68, 95% CI: 1.66–5.7). In addition, 2 studies [16,18] reported that street sweepers aged 38–47 years were more likely to develop respiratory symptoms than street sweepers whose age was below 27 years (OR = 2.17, 95% CI: 1.07–3.27) (Fig 5).

## 4. Discussion

Workplace exposure continues to be a major global public health concern, especially when it relates to respiratory diseases [1,2]. Despite the numerous studies that have been conducted to determine the prevalence of respiratory symptoms and identify contributing factors among street sweepers in LMICs, the results have been inconclusive and inconsistent. Therefore, the purpose of this study was to estimate the pooled prevalence of occupational respiratory symptoms and identify predicting factors among street sweepers in LMICs.

Overall, 47.35% (95% CI: 34.59–60.11) of street sweepers had occupational respiratory problems, according to this study. The present finding is lower than a study conducted from Ethiopia that found 51.6% [33] and 54.58% [34], as well as a global review that found 52% [35]. Workplace variations, participant differences, and the availability and appropriate usage of PPE could all be contributing factors to the potential variation. Because there are limited resources available to ensure worker safety in LMICs, occupational exposure to respiratory symptoms may increase [36].

According to the findings of this study, cough was found to be the most prevalent respiratory symptom among street sweepers at 38.37% (95% CI: 28.54–48.19). This finding is supported by other studies [34,37,38]. The possible reason for the present finding might be that, street sweepers while cleaning streets and roads, can be exposed to high levels of dust, pollutants, debris, and other airborne particles. Coughing may result from these particles irritating the respiratory system. In order to limit their exposure and lower their chance of experiencing respiratory symptoms, street sweepers should use PPE such as facemasks. Regular health examinations and appropriate training on occupational health and safety procedures are also important for safeguarding street sweepers’ respiratory health.

In this systematic review and meta-analysis study, based on a subgroup analysis, the overall estimate of respiratory symptoms among street sweepers studies conducted in Ethiopia was found to be 53.94% (95% CI: 41.21–66.68; *I*^*2*^ *= 97.25, p<0.001*), which is relatively higher than studies conducted in Egypt, Tanzania, Iran, and Nigeria. The present finding is supported by other findings [33,39]. Even though univariate meta-regressions confirmed that, the study country is not a source of heterogeneity. The possible reason for the high prevalence of respiratory symptoms in Ethiopia might be a lack of occupational health and safety training, socioeconomic level, and educational level of workers. Studies conducted in Ethiopia revealed that there is a lack of PPE and a lack of concern for the protection of the safety of workers [34,39,40].

The odds of occupational respiratory symptoms among street sweepers were 2.84 times higher for those who had had past dust exposure. This finding is supported by Asgedom [40] and Daba et al. [33]. High exposure to dust is linked to a high prevalence of respiratory disease. The main types of dust include bioaerosols, which can cause allergies and respiratory infections; vehicle dust, which has been related to Chronic Obstructive Pulmonary Disease (COPD); soil and sand dust, which can induce coughing and airway obstruction; and plant particles, which can make asthma symptoms worse. Street sweepers had significantly lower lung function than persons who are not exposed to dust, highlighting the serious health risks associated with prolonged dust exposure. Reducing these risks requires routine health examinations and the use of the proper respiratory protection equipment [32].

In this study, street sweepers who had more than five years of work experience were found to be 3.68 times more likely to develop respiratory symptoms than street sweepers who had less than five years of work experience. The present finding is supported by other studies [33,34,39,41], which implied that workers who have been in the sector for a prolonged period can develop occupational respiratory symptoms. This finding might be due to the fact that street sweepers who are exposed to high levels of dust, particulate matter, and other air pollutants can easily develop respiratory symptoms. Moreover, street sweepers with more work experience may have incompliance with the use of PPE, which leads to increased exposure to harmful airborne particles. Prolonged exposure to dust can lead to dust deposition in the respiratory system and respiratory disorders [42].

According to this systematic review and meta-analysis, street sweepers who did not use facemasks were 2.67 times more likely to develop respiratory symptoms than those who did. This finding is supported by Daba et al. [33] and Ashuro et al. [39]. Absence of occupational health and safety training and lack of PPE are the main challenges identified in many work places [40]. Evidence suggests that by using PPE appropriately, like facemasks, in work places, workers can significantly reduce occupational health-related hazards and illnesses [43].

The odds of occupational respiratory symptoms were 2.17 times higher among street sweepers whose ages were between 38–47 than those aged <27. This finding is in line with Gebremedhn and Raman [44], who state that the older age group had a longer length of service as sweepers and showed higher respiratory symptoms. The present finding also might be due to the fact that cumulative exposure to dust over many years can contribute to the development of respiratory conditions. Another possible reason might be that as the age of workers increases, their immunity level will decrease.

### 4.1. Limitation of the study

This study’s primary focus on English-language publications may cause it to miss important studies conducted in other languages that could further insight into street sweepers’ respiratory health. The included studies’ cross-sectional design may limit the ability for establishing temporary relationships. Furthermore, self-reported data were used to assess occupational respiratory problems, which could have affected the generality of the findings.

## 5. Conclusions

Occupational hazards exposure is still a major global public health concern, especially in LMICs. According to this study, occupational respiratory symptoms substantially affect nearly half of street sweepers. Street sweepers’ occupational respiratory problems were linked to a number of factors, including prior dust exposure, lack of facemask use, more than five years of work experience, and being between the ages of 38 and 47.

It is highly recommended that occupational safety policies enforce the use of PPE, such as facemasks, and establish routine health screenings to monitor respiratory health in order to address the high prevalence of respiratory problems among street sweepers. In order to reduce dust exposure, thorough training programs on the risks of dust exposure and how to use PPE should be successfully implemented.

Furthermore, measures should be taken to restrict extended exposure, and age-sensitive health interventions should be targeted at vulnerable workers. To ensure the wellbeing of street sweepers, it is also crucial to advocate for policy changes that give their health and safety top priority. It is advised that longitudinal studies be conducted in order to better understand the association between occupational exposures and respiratory health issues.

## Supporting information

S1 File Searching strategy details. (DOCX)

S2 File Study exclusion. (XLSX)

S3 File PRISMA checklist. (DOCX)

S4 File Quality assessment checklist. (DOCX)
