# Supplementary material for: Occupational respiratory symptoms and associated factors among street sweepers in low- and middle-income countries: A systematic review and meta-analysis
Source: PLoS One. 2025 Apr 9;20(4):e0320237. doi: 10.1371/journal.pone.0320237 (PMC11981149; doi:10.1371/journal.pone.0320237)
Supplement: S1 File — (DOCX) [file pone.0320237.s001.docx]

**Title: Occupational respiratory symptoms and associated factors among street sweepers in Low-income countries: A systematic review and Meta-analysis.**

| Keywords | Synonymous | Combination | Number of articles | Last search date | Electronic data base |
| --- | --- | --- | --- | --- | --- |
| Occupational | Work-related, dust exposure | Search: (((((((Occupational) OR (Work-related)) OR (dust exposure)) AND (respiratory symptoms)) OR (lung function)) AND (street sweepers)) OR (street cleaners)) AND (low and middle income countries) | 12 | 6/10/2024 | PubMed |
| Respiratory symptoms | Lung function |  |  |  |  |
| Street sweepers | Street cleaner |  |  |  |  |
| Low and middle income countries |  |  |  |  |  |
| respiratory symptoms and associated factors among street sweepers in Low-income countries |  |  | 21 | 6/10/2024 | Scientific direct |
| respiratory symptoms and associated factors among street sweepers in Low-income countries |  |  | 30 | 5/30/2024 | *Hinarie* |
| Respiratory symptoms and associated factors among street sweepers in Low-income countries |  |  | 18,100 | 6/10/2024 | Google scholar |
| Total articles | | | 18, 163 |  | |

**For PubMed search details**

((((("occupant"[All Fields] OR "occupant s"[All Fields] OR "occupants"[All Fields] OR "occupational"[All Fields] OR "occupations"[MeSH Terms] OR "occupations"[All Fields] OR "occupation"[All Fields] OR "Work-related"[All Fields] OR (("dust"[MeSH Terms] OR "dust"[All Fields]) AND ("exposure"[All Fields] OR "exposure s"[All Fields] OR "exposured"[All Fields] OR "exposures"[All Fields] OR "exposuring"[All Fields]))) AND ("signs and symptoms, respiratory"[MeSH Terms] OR ("signs"[All Fields] AND "symptoms"[All Fields] AND "respiratory"[All Fields]) OR "respiratory signs and symptoms"[All Fields] OR ("respiratory"[All Fields] AND "symptoms"[All Fields]) OR "respiratory symptoms"[All Fields])) OR ("respiratory physiological phenomena"[MeSH Terms] OR ("respiratory"[All Fields] AND "physiological"[All Fields] AND "phenomena"[All Fields]) OR "respiratory physiological phenomena"[All Fields] OR ("lung"[All Fields] AND "function"[All Fields]) OR "lung function"[All Fields])) AND (("street"[All Fields] OR "street s"[All Fields] OR "streets"[All Fields]) AND ("sweeper"[All Fields] OR "sweepers"[All Fields]))) OR (("street"[All Fields] OR "street s"[All Fields] OR "streets"[All Fields]) AND ("cleaner"[All Fields] OR "cleaners"[All Fields]))) AND ("developing countries"[MeSH Terms] OR ("developing"[All Fields] AND "countries"[All Fields]) OR "developing countries"[All Fields] OR ("low"[All Fields] AND "middle"[All Fields] AND "income"[All Fields] AND "countries"[All Fields]) OR "low and middle income countries"[All Fields])

**Translations**

**Occupational:** "occupant"[All Fields] OR "occupant's"[All Fields] OR "occupants"[All Fields] OR "occupational"[All Fields] OR "occupations"[MeSH Terms] OR "occupations"[All Fields] OR "occupation"[All Fields]

**dust:** "dust"[MeSH Terms] OR "dust"[All Fields]

**exposure:** "exposure"[All Fields] OR "exposure's"[All Fields] OR "exposured"[All Fields] OR "exposures"[All Fields] OR "exposuring"[All Fields]

**respiratory symptoms:** "signs and symptoms, respiratory"[MeSH Terms] OR ("signs"[All Fields] AND "symptoms"[All Fields] AND "respiratory"[All Fields]) OR "respiratory signs and symptoms"[All Fields] OR ("respiratory"[All Fields] AND "symptoms"[All Fields]) OR "respiratory symptoms"[All Fields]

**lung function:** "respiratory physiological phenomena"[MeSH Terms] OR ("respiratory"[All Fields] AND "physiological"[All Fields] AND "phenomena"[All Fields]) OR "respiratory physiological phenomena"[All Fields] OR ("lung"[All Fields] AND "function"[All Fields]) OR "lung function"[All Fields]

**street:** "street"[All Fields] OR "street's"[All Fields] OR "streets"[All Fields]

**sweepers:** "sweeper"[All Fields] OR "sweepers"[All Fields]

**street:** "street"[All Fields] OR "street's"[All Fields] OR "streets"[All Fields]

**cleaners:** "cleaner"[All Fields] OR "cleaners"[All Fields]

**low and middle income countries:** "developing countries"[MeSH Terms] OR ("developing"[All Fields] AND "countries"[All Fields]) OR "developing countries"[All Fields] OR ("low"[All Fields] AND "middle"[All Fields] AND "income"[All Fields] AND "countries"[All Fields]) OR "low and middle income countries"[All Fields]
