# Supplementary material for: Occupational respiratory symptoms and associated factors among street sweepers in low- and middle-income countries: A systematic review and meta-analysis
Source: PLoS One. 2025 Apr 9;20(4):e0320237. doi: 10.1371/journal.pone.0320237 (PMC11981149; doi:10.1371/journal.pone.0320237)
Supplement: S4 File — (DOCX) [file pone.0320237.s004.docx]

**Results of JBI Quality Assessment**

| Studies | Clear eligibility criteria | Description of study subject and study setting | Valid and reliable method to measure the exposure | Standard criteria used for measurement of the condition | Identification of confounding factors | Develop of strategies to deal with confounding factors | Valid and reliable method to measured outcomes | Appropriate statistical analysis | Total score out of 8 | Level of bias |
| --- | --- | --- | --- | --- | --- | --- | --- | --- | --- | --- |
| Worede et al. | Unclear | No | Yes | Yes | N/A | Yes | Yes | Yes | 6 | Low |
| Tamene et al. | Unclear | Yes | Yes | Yes | Yes | Yes | Yes | Yes | 7 | Low |
| Manaye et al. | Unclear | Unclear | Yes | Yes | N/A | Yes | Yes | Yes | 6 | Low |
| Wubet | Unclear | Yes | Yes | Yes | Yes | Yes | Yes | Yes | 7 | Low |
| Beyene | Unclear | Yes | Yes | Yes | N/A | No | Yes | Yes | 6 | Low |
| Eneyew et al. | Unclear | No | Yes | Yes | N/A | Yes | Yes | Yes | 6 | Low |
| Stambuli | No | Yes | Yes | Yes | N/A | No | Yes | Yes | 6 | Low |
| Mostafa et al. | Yes | Yes | No | Yes | Yes | No | No | Yes | 4 | Moderate |
| Habybabady et al. | Unclear | Yes | No | Yes | Yes | No | No | Yes | 4 | Moderate |
| Nku et al. | Unclear | Yes | Yes | No | No | No | Yes | No | 3 | Moderate |
